# Evaluation of oat β-glucan-marine collagen peptide mixed gel and its application as the fat replacer in the sausage products

**DOI:** 10.1371/journal.pone.0233447

**Published:** 2020-05-22

**Authors:** Rui Fan, Dan Zhou, Xueli Cao

**Affiliations:** 1 Beijing Advanced Innovation Center for Food Nutrition and Human Health, Beijing Technology & Business University, Beijing, P. R. China; 2 Department of Nutrition and Food Hygiene, School of Public Health, Peking University, Beijing, P. R. China; 3 School of Life Science and Technology, Beijing University of Chemical Technology, Beijing, P. R. China; University of Hyderabad, INDIA

## Abstract

The food industry is currently shown the concern with low-fat products. This study aims to evaluate the properties of oat β-glucan(OG)-marine collagen peptide (MCP) mixed gels induced by high pressure at different ratios, pressures, pH levels and the superiority of application in the sausage. The results indicated that the typical gel with high levels of hardness, cohesiveness, springiness, and chewiness, as well as high water holding and oil adsorption capacities was formed using the OG/MCP ratio of 10:1 under 400 MPa at pH 6.0. The mixed gel replacing with 50% fat significantly increased the springiness and chewing(*P*<0.05), and sausages with 80% mixed gel were significantly juicier than that of the control sausage(*P*<0.05). Therefore, OG-MCP mixed gel could be used in the reformulation of low-fat meat products to enhance their safety and nutritional value.

## 1. Introduction

It is well known that the intake of high fat diet has been associated with increased risk of obesity, coronary heart disease, hypertension and insulin resistance etc. [1]. The World Health Organization has proposed limiting total fat intake to 30% of total calorie intake [2]. Therefore, the total content and percentage of calories provided by fat are main concerns in researching and development the new healthy food product, which requires extensive research on low-fat foods, particularly relevant to the meat industry [3].

The meat industry has taken a great interest in manufacturing low-fat meat food products. In the early years, several studies have addressed the low-fat products through replacement of fat with water [4], monounsaturated oils [5], carbohydrate-based and protein-based fat substitutes [6,7], as well as modification of fatty acids [8]. Reducing fat in meat products presented challenges in appearance, flavor, and texture, which affect consumer acceptability [9]. Accordingly, several modifications have been introduced to mitigate the negative effects of reducing the fat level in meat products by conferring desirable texture and, enhancing their water-holding capacity. Many reports have shown that some carbohydrates, fibers, and proteins have reduced the cooking loss, formulation cost and enhancing the texture of meat products [10,11].

Oat and its constituents have gained attention for their uses in low-fat products. Different types of oat fiber have been used in several meat products [12]. Nutrim10^®^_,_ a dietary fiber product, has recently been produced as an alternative source of the soluble fiber β-glucan. It has also been used as a cream and fat replacer, nutrifiber, and texturizer [12]. However, oat fiber can lead to cooking loss, and its effects on the quality of low-fat meat foods remain inconclusive [13]. Recent reports have indicated that using hydrocolloids as fat substitutes enable fat reduction while providing lubrication and mouth-feel attributes that simulate the organoleptic qualities of fat [12]. Studies have been conducted on adding different kinds of protein and polysaccharide gels to meat products, which result in their diverse characteristics [14,15]. However, the use of oat β-glucan gel as a fat replacer in meat foods has rarely been reported.

The physicochemical characteristics of fat replacers need to be considered because they could play the main role of the quality properties of reformulated products. The properties of β-glucan are evidently influenced by high pressure; thus, high pressure can be potentially used to induce the β-glucan gel formation, which could modify gels as fat replacers [16]. Meanwhile, β-glucan gels with different molecular weights have showed the variability in their properties, such as viscous [17]. Thus, on the basis of our previously published report [16], the mixed gel with different molecular weight, β-glucan and marine collagen peptide, was chosen as the fat replacer in this study.

No references regarding the characteristics of β-glucan-peptide mixed gels used as animal fat substitutes are thus far known. The study aimed to evaluate the physicochemical characteristics of oat β-glucan(OG)-marine collagen peptide (MCP) mixed gel induced by high pressure. It also intends to determine the suitability of a fat replacer in sausages depending on how well it reproduces the characteristics of the meat matrix, including color, textural properties, or cooking properties. The study can thus contribute to the utilization of a more suitable fat replacer in the low-fat meat foods.

## 2. Materials and methods

### 2.1 Materials

Oat β-glucan (food-grade, purity>85%) with a molecular weight of 2.0×10^5^ Da was purchased from Guangzhou Zhongkang food co. LTD (Guangdong, China). Marine collagen peptide (food-grade, purity>85%) with a molecular weight of 3,000 Da was obtained from SEMNL food co. LTD (Beijing, China). The pork tenderloin and spices were bought from the local market, and the pork backfat was obtained by removing from pork. The additives (food-grade) were purchased from Hebei Chuangzhiyuan biotechnology co. LTD (Shijiazhuang, China). All other chemicals used were of analytical grade.

### 2.2 Properties of oat β-glucan and marine collagen peptide mixed gels

#### 2.2.1 Preparation of mixed gels

On the basis of the previously experiments [16], the oat β-glucan (OG) (6g) and the specific amount of marine collagen peptide (MCP) were weighted and then dissolved in distilled water (pH≈6) or a buffer solution at pH 3.0 (10 mM glycine–HCl buffer) to prepare solutions with OG concentration of 12% (w/v) at the certain OG/MCP ratios (8:1, 8:3, 8:5,10:1,10:3, 10:5, 6:1, 6:3, 6:5, w/w). i.e. the amount of OG and MCP was 6+0.75g, 6+2.25g, 6+3.75g, 6+0.6 g, 6+1.8 g, 6+3 g, 6+1 g, 6+3 g, 6+5 g. All samples were magnetically stirred at 25°C for 4 h. The β-glucan and marine collagen peptide mixed solution was subjected to high-pressure at 400 or 500 MPa by using the HPP L2-700/1 ultrahigh pressure device (Tianjin Huatai Senmiao Biotechnology and Technique Co. Ltd., Tianjin, China). The operation procedure was followed the method from Fan’s report [16].

#### 2.2.2 Rheological measurements of OG-MCP mixed gels

Rheological measurements of OG-MCP mixed gels were carried out based on the method from Fan’s report by the dynamic oscillatory shear tests using a controlled stress rheometer AR-1500ex (TA Instruments, Delaware, USA) [16].

#### 2.2.3 Texture profile analysis of OG-MCP mixed gels

According to the method reported by Fan, textural profile analysis of the OG-MCP mixed gels was assessed using the TA-XT Plus Texture Analyzer (Stable Micro Systems, Godalming, UK) [16].

#### 2.2.4 The water holding capacity and oil adsorption capacity

The determination of water holding capacity (WHC) was according to Li's method with the minor modifications [18]. The OG-MCP mixed gels (about 3g) was centrifuged with 8000 r/min for 30 min, then the surface water was removed, and the total weight of centrifuge tube and mixed gel was weighed before and after centrifugation, until the difference between the two consecutive runs was less than 0.05 g. WHC was described as the Eq (1).

$$WHC\;(\%)=\frac{W_{1}‐W_{0}}{W_{2}‐W_{0}}\times 100\%$$ Eq (1)

where W_1_ is the total weight (g) of centrifuge tube and mixed gel after centrifugation, W_2_ is the initial total weight (g) of centrifuge tube and mixed gel, W_0_ is the weight (g) of centrifuge tube.

The oil adsorption capacity (OAC) was determined was according to Zheng's method with the minor modifications [19]. The OG-MCP mixed gels (about 3g) was mixed with soybean oil in a centrifugal tube and left for 1 h at room temperature. The mixture was then centrifuged at 8000 r/min for 5 min, and the supernatant was decanted and the pellet recovered by filtration through a linen mesh. OAC was described as the Eq (2).

$$OAC\;(\%)=\frac{W_{1}-W_{0}}{W_{0}}\times 100\%$$ Eq (2)

where W_1_ is the pellet weight (g), W_0_ is the initial weight mixed gel (g).

### 2.3 The application of OG-MCP mixed gels in the sausages

#### 2.3.1 Preparation of low-fat sausage samples

In the preliminary experiments, pork content and fat/lean ratio in the control sausage (CS) were determined based on sensorial acceptability. The low-fat sausage (LFS) samples LFS (80), LFS (50), and LFS(20) were prepared using the ingredients listed in Table 1. The OG–MCP mixed gel was used to substitute 80%, 50%, and 20% of pork backfat in LFS (80), LFS (50), and LFS (20), respectively.

**Table 1:** All ingredients (g) of 100 g raw batter.

Ingredient	CS	LFS (80)	LFS (50)	LFS (20)
Pork tenderloin	60	60	60	60
Pork backfat	25	5	12.5	20
OG-MCP mixed gel	-	20	12.5	5
Salt	3	3	3	3
Ice/water	10	10	10	10
Spices and additives^a^	2	2	2	2

^a^ The compositions of spices and additives included sugar(1.5g/100g raw batter), garlic (0.1g/100g raw batter), pepper(0.02g/100g raw batter), chili powder (0.1g g/100g raw batter), aginomoto (0.06g/100g raw batter), D-sodium erythorbate (0.02 g/100g raw batter), sodium pyrophosphate (0.15 g/100g raw batter), NaNO_2_(0.05g/100g raw batter).

Sausage batters were prepared and cooked using the methods described in previous reports, with minor modifications [20,21]. First, pork tenderloin and backfat/mixed gel were minced into particles measuring 5 mm. Second, the minced meat was placed in a mixer to which half of ice/water, salt, and additives were added. The mixture was then stirred for 10 min to obtain meat paste. Third, the meat paste was placed into the container for curing at 4°C for 24 h, with relative humidity at 80%. The meat paste was then transferred to a chopper mixer and chopped for 1 min. Residual ice/water and spices were subsequently added into the meat paste and then stirred for 3 min. The temperature during the aforementioned processes was limited to below 10°C. The meat paste was manually stuffed in a casing measuring 22 mm in diameter. The sausage samples were finally cooked for 30 min in a steam chamber until the center temperature of the sausage samples reached 80°C. The samples were cooled immediately in a cold water and then stored at 4°C until measurement.

#### 2.3.2 Composition of the sausage samples

In accordance with AOACT, the components of the sausage samples including moisture, carbohydrate, fat, crude protein and ash were determined [22].

#### 2.3.3 Cooking loss and water-holding capacity

Cooking loss was measured using the method described by Mohammadi [2]. The sausage samples were weighed, cooked, chilled for 24h at 0–4°C, and then reweighed. Cooking loss was calculated using as the percentage difference between the weights before and after cooking.

#### 2.3.4 Sensorial analysis of the sausage

The sausage samples were evaluated by 15 untrained assessors consisting of students and faculty members in our institution. Sensorial analysis was conducted by triangle testing using the method described in the study by Mendoza with some modifications [10]. The assessors measured the differences between low-fat and normal sausage samples on the basis of the following attributes on a non-structured 10cm hedonic scales: color, flavor, texture, and taste (0 = very unpleasant and 10 = very pleasant), overall palatability (0 = dislike extremely and 10 = like extremely), juiciness (0 = very dry and 10 = very juicy).

#### 2.3.5 Color measurements

The sausage color was measured with a tristimulus reflectance colorimeter (Minolta CR-300, Minolta Corp., USA). Color was expressed as the CIE-L*, a* and b* scales. The letter L* determines the change in the brightness of the test sample on a scale of 0 to 100 (the higher the value of L, the sample is brighter), the letter a* represents green (a* = -100) and red (a* = 100), while the letter b* represents blue (b* = -100) and yellow (b* = 100). The device was calibrated before the test on the white standard. Each sample was placed on a white table, L*, a*, b* and CIELAB color measurements were then taken for three times. The light pulses were timed to move the colorimeter to three locations of each sample.

### 2.4 Ethics statement

The ethics statement wasn’t approved by any agency, although human participated the sensorial evaluation in this study. Because these participants just tasted the sausages without any types of health-related research, which is low-risk research. Before starting the study, the methods and main points of sensory evaluation were told by our technical staff, which were easily understood and mastered due to participants’ majors related to food chemistry, meanwhile, the informed consent was read and signed by the participants.

### 2.5 Statistical analysis

The results were presented as mean±standard deviation, and the means were calculated using three different methods of determination. The means were compared by using ANOVA, and the significance(*P*<0.05) of the differences was determined using the Duncan multiple-range test in SPSS 18.0 (SPSS Inc., Chicago, USA).

## 3. Results and discussion

### 3.1 Rheological behavior of OG–MCP mixed gels

**Fig 1:**
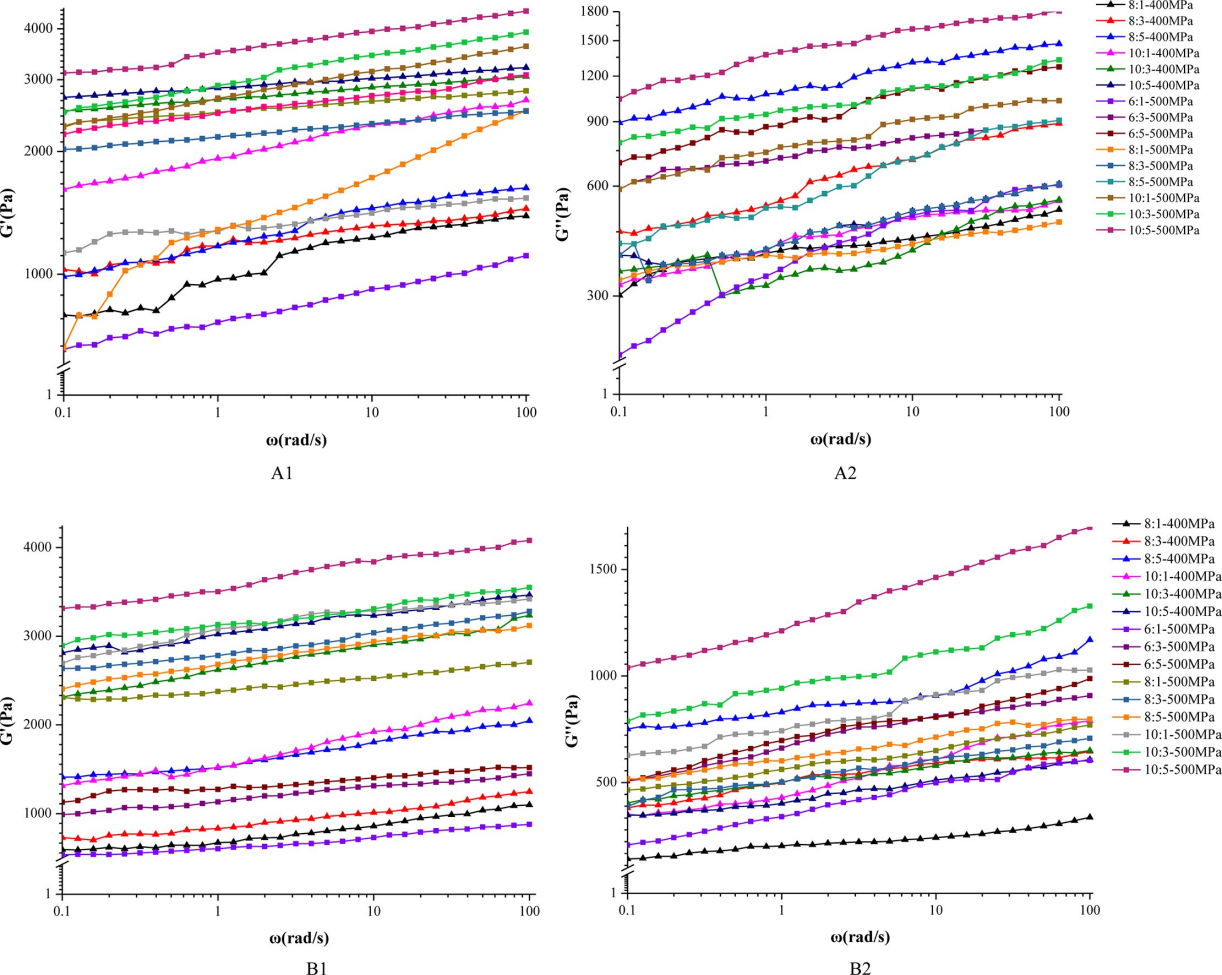
Frequency sweep curves (G' and G'') of β-glucan/ marine collagen peptide (OG-MCP) mixed gels at different pHs (A1 and A2, pH 6; B1 and B2, pH 3).

Fig 1 presents the frequency sweep curves of OG–MCP mixed gels. The OG/MCP ratio and pressure exerted evident effects on gel formation. A typical gel is formed when G' is considerably larger than G'', and the two value rarely depend on frequency, the curve of G' is parallel with the curve of G'' [16,23,24]. In Fig 1, G' was larger than G'' under all parameters; meanwhile, the mixed gels prepared with OG/MCP ratios of 10:3 and 10:5 under 400 MPa and OG/MCP ratios of 8:1 and 8:3 under 500 MPa at pH 6.0 were typical gels. In addition, a true elastic gel network can form when G' is at least 10 times greater than G'', and each value rarely depends on frequency [25]. Therefore, the mixed gel with the OG/MCP ratio of 10:3, induced at 400 MPa, as well as the mixed gel with the OG/MCP ratio of 8:1, induced at 500 MPa, formed typical gels with a true elastic gel network.

We also observed an unparallelled curve between G' and G'' at ratios of 8:1 and 10:1 under 400 MPa(Figs A1 and A2, respectively), indicating that the gels formed with these ratios was not the typical gel. With an increase in β-glucan ratio, more β-glucan was be added, which could lead to numerous entanglements. Additional entanglements could limit the mobility of β-glucan and block the rearrangement into larger aggregated structures, which prevented gel from forming the network structure [26]. With the OG/MCP ratio set to 10:3 under 400 MPa, a typical gel was formed, demonstrating their stable network structure, this finding agreed with other study [18]. The low-molecular-weight compounds could serve as linker molecules for the high-molecular-weight β-glucan [17,27]. In Figs A1 and A2, G' and G'' increase with higher frequency when pressure is increased, exhibiting a typical viscoelastic characteristic. Numerous studies have reported on hydrogels produced from aqueous solutions containing linear or branched PEG macromolecules via chemical crosslinking [28]. Noncovalent interactions provide an alternative method for crosslinking. The ability of a peptide to form non-covalent interactions with both the polymer and other peptides may affect the polymer–peptide physical properties, which can subsequently influence its applicability as an injectable gel[29].

We also observed that G' and G'' were larger at pH 3.0 than at pH 6.0 regardless of the conditions. β-glucan (a soluble anionic polysaccharide) and MCP may form a gel at pH 3.0, close to the isoelectric point (pI≈3.2) of MCP, which could result from electrostatic interaction.

In Fig 1B, G' is larger than G'' under all treatment conditions; meanwhile, the typical gels were prepared with OG/MCP ratios of 10:1, 10:3, and 10:5 under 400 MPa; those with ratios of 8:3 were under 500MPa at pH 3.0. The gels with a true elastic gel network were those prepared with OG/MCP ratios of 10:5 and 8:3 under 400 and 500 MPa, respectively.

### 3.2 The textural properties of OG-MCP mixed gels

**Fig 2:**
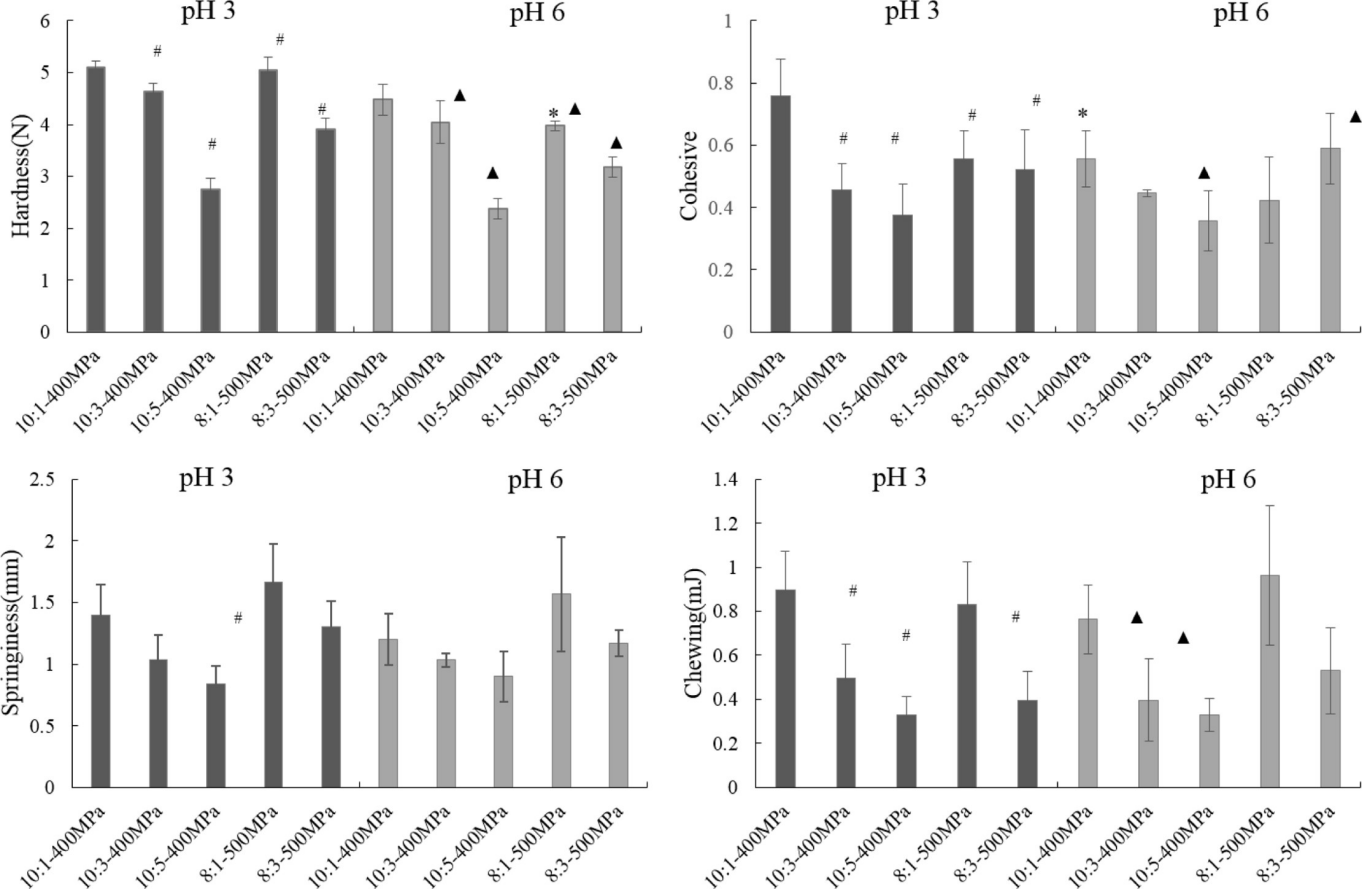
Textural property of OG-MCP mixed gels at different conditions (mean±SD, n = 6). * compared with the same preparation parameter between pH 3 and pH 6 (*P* < 0.05); # compared with 10:1-400MPa at pH3 (*P* < 0.05); ▲ compared with 10:1-400MPa at pH6 (*P <* 0.05).

Texture is an important property that determines the organoleptic quality of gels. The effects of pressures, pH, and ratios on the textural properties of the mixed gels are illustrated in Fig 2. Ratio and pressure evidently influenced the textural properties (*P*<0.05), whereas pH only slight affected the same(*P*>0.05). Fig 2 shows that hardness, springiness, cohesiveness, and chewiness decreased when the OG/MCP ratio is reduced; meanwhile, hardness and springiness increase when pressure is increased. A previous report had approved that more than 400 MPa could induce forming starch gels [30]. In addition, high-molecular-weight β-glucan gels formed a denser network, and MCP with low-viscosity could move easily [31]. Thus, high hardness was attributed to the denser networks of mixed gels with higher β-glucan ratio and pressure, reinforcing the intermolecular interaction of β-glucan and MCP, thereby increasing hardness and springiness [18,32]. This occurrence was consistent with the change in G'.

### 3.3 The WHC and OAC of OG-MCP mixed gels

**Fig 3:**
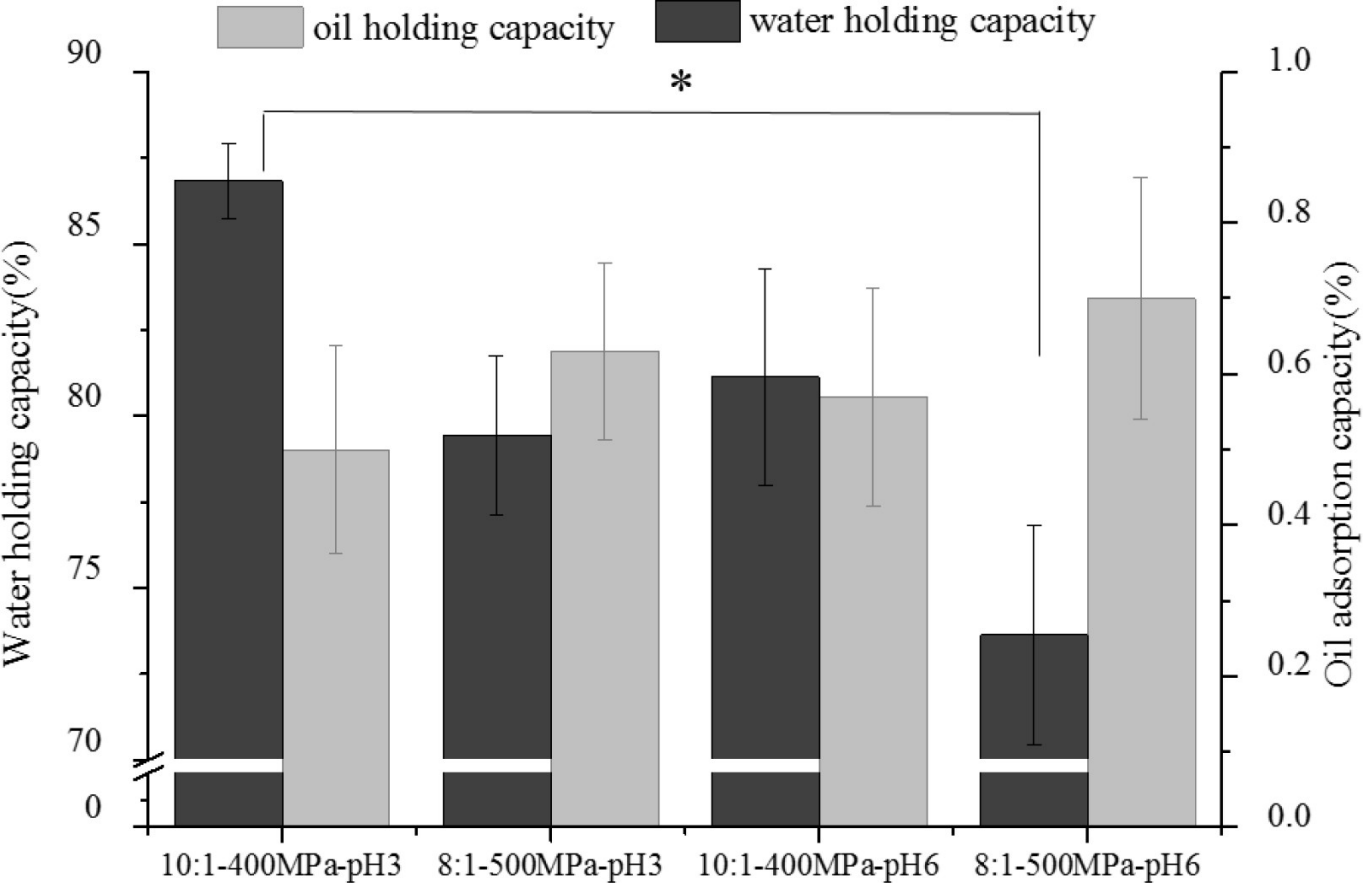
The changes of WHC and ODC of OG-MCP mixed gels. * compared with 10:1-400MPa at pH3 (*P* < 0.05).

WHC, the capacity of raw materials to retain water, is directly related to the texture, structure, and state of the gel [33]. Fig 3 shows the changes in the WHC of the different OG–MCP mixed gels; the mixed gels with higher β-glucan content had the larger WHC value at the same pH. This finding was consistent with the G' value in Fig 1. The WHC of a gel was determined by both the microstructure and stiffness of the gel [34]. It was attributed to the increase in β-glucan, which strengthened the intermolecular interaction between β-glucan and MCP and between β-glucan and water. The WHC of the mixed gel with the same ratio under the same pressure at pH = 3 was higher than that at pH = 6, but the difference was not significant(*P*>0.05).

OAC is the binding of fat by a nonpolar amino acid present in the side chains of proteins [35]. As shown in Fig 3, the mixed gel with a higher MCP content showed a higher OAC at the same pH. The OAC of the mixed gel prepared at pH 6 was higher than that at pH = 3. As the pH of the compounds was decreased to its isoelectric point, the magnitude of electrostatic repulsive forces between the protein and the peptide decreased, and hydrophobic attraction prompts proteins to aggregate [36]. However, the change in OAC at different pH levels was not evident (*P*>0.05). Thus, with the actual operating conditions for sausage considered, the following conditions were regarded as optimal: pH = 6; OG/MCP ratio of 10:1; and pressure = 400 MPa.

### 3.4 The sausage properties evaluation

#### 3.4.1 The composition of sausages

The composition of sausages shows in Table 2. As the ratio of fat replacement increases, the fat content in the sausages decreases significantly from 22.26 to 7.08%(*P*<0.05). A similar behavior was observed in ash content; with an increase in the ratio of the fat replacer, the carbohydrate increased significantly(*P*<0.05). The fat contents in LFS (80) were less than 10%, and the sausages were considered as low-fat [2]. Much of the moisture was shown in the low-fat sausages owing to the good WHC of the OG–MCP mixed gel. The carbohydrate content in the sausages containing the fat replacers LFS (50) and LFS (80) was significantly higher (*P<*0.05) than that in other sausages because the fat replacers were carbohydrate-based components. Similar results were found in other studies [11,37].

**Table 2:** Proximate composition of sausages formulated with varying levels of fat replacer.

Chemical component(g/100g)	Fat	Protein	Carbohydrate	Ash	Moisture
CS	22.26(2.34)	19.33*(2.01)	1.63(0.28)	2.34(0.23)	62.34(3.12)
LFS(20)	15.22(1.22) *	17.88(1.89)	2.20(0.49)	2.20(0.33)	64.74(1.82)
LFS(50)	11.74(2.04) *	18.58(1.78)	4.01(0.78)*	2.11(0.13)	67.30(2.12)*
LFS(80)	7.08(1.89)*	20.99(1.01)	6.42(0.87)*	2.01(0.11)	70.02(2.12)*

* compared with CS (*P* < 0.05)

#### 3.4.2 The textural properties of sausages

**Fig 4:**
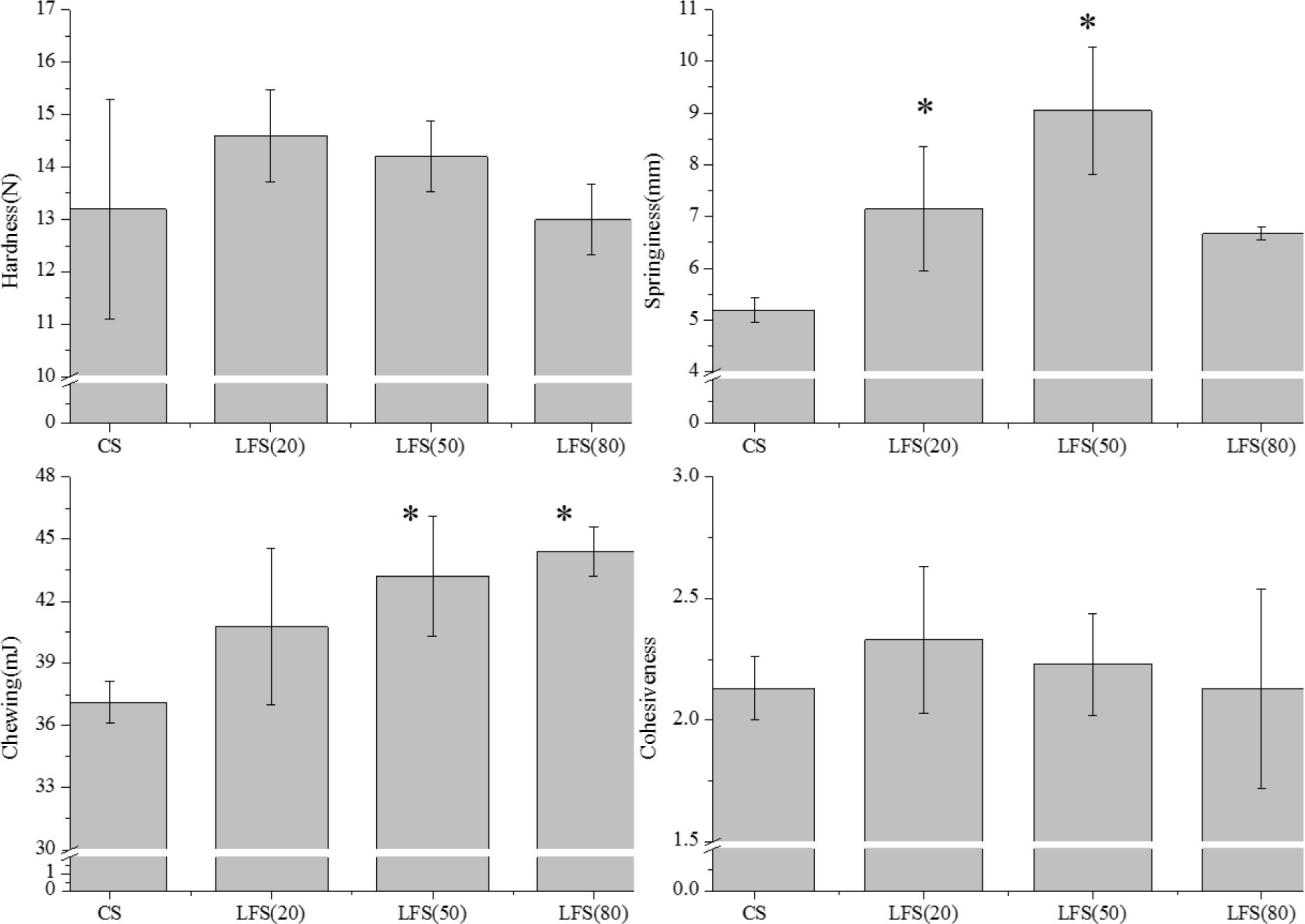
The textural properties of cooked sausages. * compared with CS (*P* < 0.05).

Fig 4 presents the effects of fat replacers on the textural properties of the sausages. Springiness represents the extent of recovery of sausage height [12]. The addition of fat replacers significantly affected the texture of the sausage. With an increase in fat replacer, the hardness, springiness, and cohesiveness of sausage decreased but not chewiness; however, the hardness and cohesiveness of the different sausage samples showed no significant difference(*P*>0.05). These results were consistent with previous reports [38]. Desmond (1998) reported that beef burgers containing oat fiber exhibited a reduction in hardness [39]. Carballo (1996) found the amount of oat, starch, and egg white brought the slight differences in cohesiveness of bologna sausages [40]. The low-fat sausage with 20% of its backfat replaced exhibited the highest levels of hardness and cohesiveness; meanwhile, the mixed gel replacing with 50% fat significantly increased the springiness and chewing(*P*<0.05). A decrease in the hardness of sausage samples was due to texture-modifying ingredients which might help absorb and retain moisture to increase WHC [41]. In addition, the presence of texture-modifying extenders, rather than the water binding property of the extenders, may reduce binding among the proteins [42]. The chewiness of low-fat sausages was higher than that of the CS, and the higher addition of fat replacer added showed significant differences among the CS.

#### 3.4.3 The cooking properties of sausages

**Fig 5:**
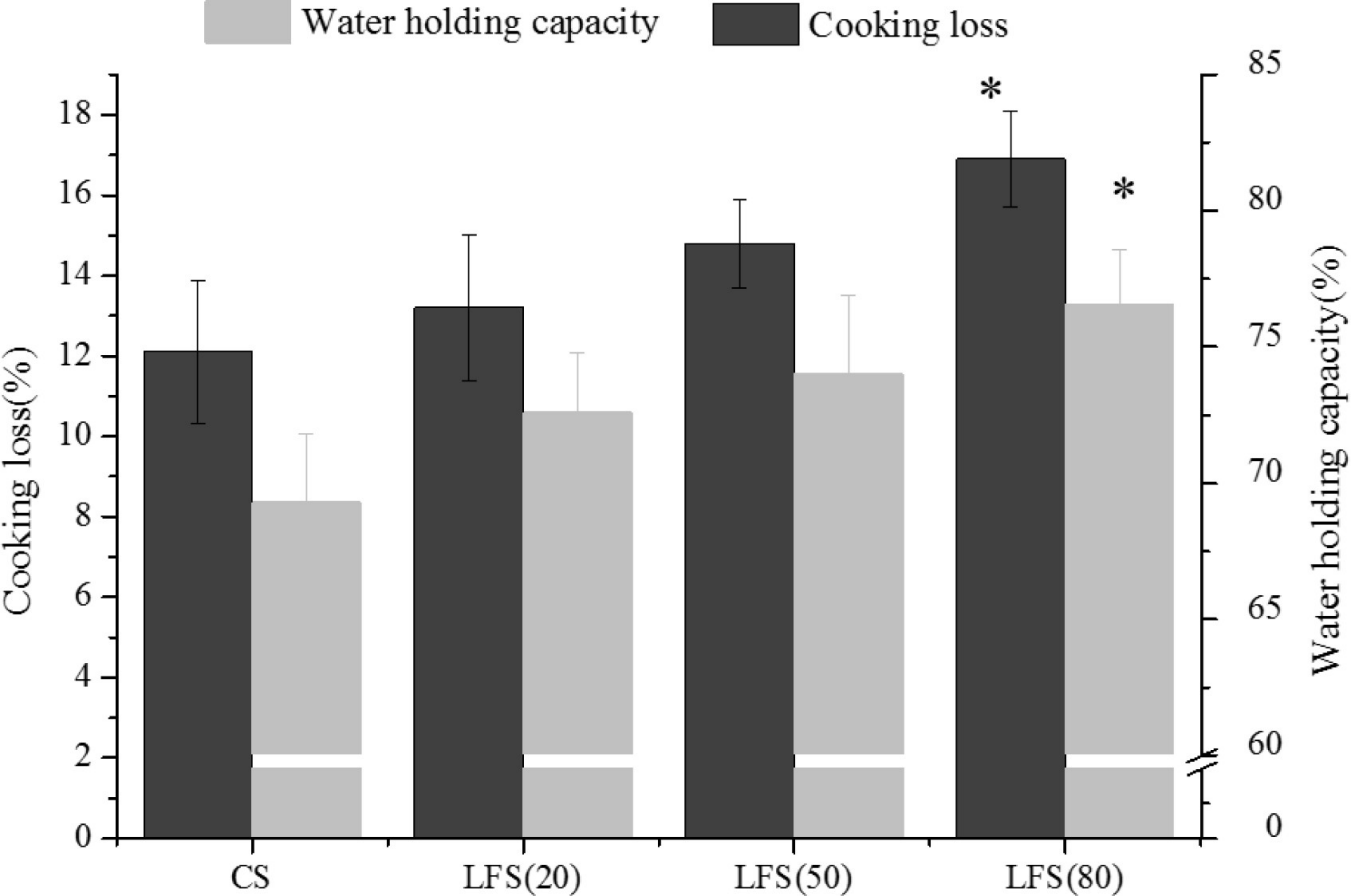
The water holding capacity and cooking loss of the sausages. * compared with CS (*P* < 0.05).

The WHC and cooking loss of the sausages are presented in Fig 5. The WHC increased as the percentage of fat replacer increased. The WHC of all low-fat sausages was higher than that of the CS; the WHC of LFS (80) was significantly higher than that of the CS (*P*<0.05). The results were consistent with the other reports [43,12]. Piñero (2008) also reported that the carbohydrate could lead to WHC increasing, which was due to the type and content of protein and carbohydrates [11]. The ability of oat hydrocolloidal fiber(β-glucan) to create a tridimensional matrix, which can help holding not only water but also fat to prevent their loss during cooking. This property was due to the swelling of polysaccharide embedded in the protein gel matrix, structural formation during heating, and abundant intermolecular bridges[11]. Meanwhile, increasing the peptide could increase the number of locations capable of interacting during heating in polypeptide chains [44].

The CS and the sausages with low fat replacer contents showed low cooking loss. LFS (80) showed the highest cooking loss, which was significantly higher than CS (*P*<0.05). A similar behavior was reported in a previous study [42,45,21]. However, only small differences in cooking loss (27–29%) owing to fat content (5–29%) were found in breakfast sausages [46]. With considerable amounts of oat added and extended cooking time, oat starch was more likely to form gels. Although gelatinization leads to increased hydration of starch, it does not provide increased resistance to loss of moisture during cooking. This finding may be attributed to the pregelatinization of β-glucan prior to cooking; internal pressures from meat protein network changing may drive water away from the mixed gel [42].

#### 3.3.5 The sensory evaluations of sausages

Table 3 shows the color characteristics of the manufactured sausages. The color of CS had obviously higher(*P*<0.05) L* and a* values and lower b* value than those of low-fat sausages. With an increase in the fat replacer ratio to 50%, L* and a* significantly decreased(*P*<0.05), whereas b* increased. Fat replacers were generally darker and yellower than backfat, increasing b* and decreasing L* in low-fat sausages. The contribution of backfat to the meat color depends on the characteristics of the product. Whereas fat and lean in sausage can be distinguished by the naked eye, the further structure of meat when it is broken down becomes more difficult to distinguish until the distinction is replaced by a more general perception of color [14]. Fat content and the fat replacer affect the color parameters of the meat product. Thus, decreasing fat content lead to darker colour [40].

**Table 3:** Colour parameters of sausages formulated with varying levels of fat replacer.

Sausages	L*	a*	b*
CS	79.54±1.38	4.47±0.38	7.65±0.68
LFS(20)	78.12±1.28	4.26±0.28	8.21±0.88
LFS(50)	75.8±2.22*	3.92±0.48	9.46±0.98*
LFS(80)	72.12±1.41*	3.26±0.42*	10.15±1.02*

* compared with CS (*P* < 0.05)

**Fig 6:**
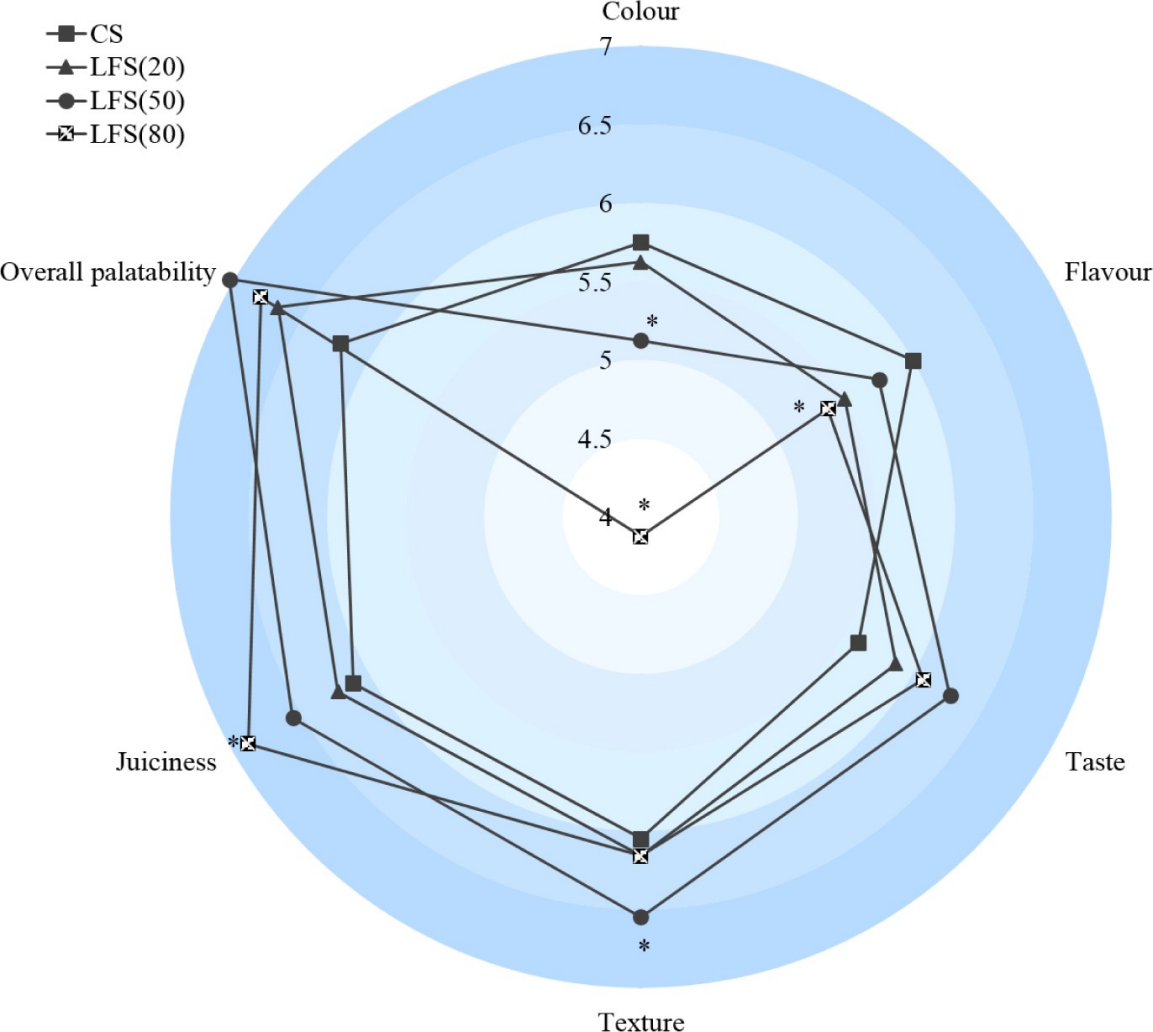
The effect of fat replacer on sensory attributes of sausages. * compared with CS (*P* < 0.05).

Fig 6 shows the results of the sensory analysis. The various concentric circles imply the different score scale range from 4 to 7, which represented the farther from the center, the higher the score. Sausages with 80% fat replacers were significantly juicier than the CSs(*P*<0.05), which could be attributed to the increased WHC of the OG–MCP mixed gel. These findings agreed with that of Desmond [39]. Pszczola (1991) reported that oat fiber can retain water and prevent meats from drying during cooked [43]. The tenderness of the sausage products was improved with the addition of the fat replacer owing to the higher WHC and OAC and better texture characteristics of the fat replacer. Several studies reported that the addition of starch, polysaccharides, or non-meat proteins in muscle-based food could bring an acceptable product. Huffman reported that adding 1.5% carrageenan into lean-pork sausage could increase the juiciness and tenderness [47]. Chang (1997) showed water and oat bran could significantly influence the juiciness and graininess, and the optimal formulation for frankfurters had 2% added oat bran [48]. The taste and overall palatability of low-fat sausage was comparable to those of the control (*P*>0.05), whereas the flavour and color of low-fat sausages had lower sensory evaluation score than those of the control. These results indicated that compared with the factors influence the contribution of fat to the appearance, those relative proportions of the different types of fat also need to be considered. Consequently, the effects of reformulation would be elucidated, and better fat analogs could be formulated for use in low-fat meat processing [14].

## 4. Conclusion

The preparation of the OG–MCP mixed gel by high pressure was optimized in this study. The typical gel as the fat replacer was formed with the OG/MCP ratio of 10:1 under 400MPa at pH 6.0. Given these parameters, the OG–MCP exhibited high levels of hardness, cohesiveness, springiness, and chewiness, as well as high WHC and OAC. The preparation of low-fat sausage with the fat replacer was determined, and their use in sausage samples led to increased WHC and enhanced sensory characteristics than those of the CS. This OG–MCP mixed is particularly suitable as a pork backfat replacer in fat-reduced sausage, which can improve the nutritional value of the product.
